# ICG clearance as an indicator of augmented hepatic clearance and subtherapeutic drug concentrations in septic patients

**DOI:** 10.1186/s12871-025-03534-9

**Published:** 2025-12-10

**Authors:** Ying Xu, Xiaoting Wu, Ming Yan, Wenkui Yu, Danjiang Dong, Chen Qu, Ning Liu

**Affiliations:** 1 Department of Intensive Care Unit, Nanjing Drum Tower Hospital, The Affiliated Hospital of Medical School, Nanjing University , Zhongshan Road 321#, Gulou District, Nanjing, Jiangsu China; 2 Geriatric Medicine Department, The Second Affiliated Hospital of Nanjing Medical University, Jiangjiayuan Road 121#, Gulou District, Nanjing, Jiangsu China

**Keywords:** Indocyanine green, ICG-R15, ICG-PDR, Augmented hepatic clearance, Subtherapeutic drug concentration, Sepsis

## Abstract

**Purpose:**

To assess indocyanine green (ICG) clearance as a quantitative indicator of augmented hepatic clearance (AHC) in septic patients and to evaluate its impact on subtherapeutic antibiotic concentrations and 28-day mortality.

**Methods:**

This prospective observational study enrolled 93 septic patients admitted to the intensive care unit (ICU), from whom 113 ICG clearance tests were obtained. Patients were stratified into three groups based on ICG retention rate at 15 min (ICG-R15): augmented (AHC, ICG-R15 < 6%), normal (NHC, 6% ≤ ICG-R15 ≤ 12%), and impaired hepatic clearance (IHC, ICG-R15 > 12%). Trough concentrations were measured for the following antibiotics: imipenem/cilastatin, piperacillin/tazobactam, voriconazole, linezolid, vancomycin, cefoperazone/sulbactam, and teicoplanin. Correlations between ICG clearance parameters (ICG-R15 and ICG plasma disappearance rate [PDR] ) and antibiotic concentrations were analyzed. Multivariate logistic regression and ROC analysis were performed to identify independent predictors of subtherapeutic concentrations.

**Results:**

Nearly half of the cohort (49.5%, 46/93) presented with AHC. The 28-day mortality rate was highest in the IHC group (48.4%), with no significant difference between the AHC (10.9%) and NHC (12.5%) groups. Trough concentrations of hepatically or partially cleared antibiotics (voriconazole and linezolid) showed strong correlations with ICG-R15 (*r* = 0.679 and *r* = 0.626, respectively) and ICG-PDR (*r* = -0.673 and *r* = -0.629, respectively; all *p* < 0.01). No significant correlations were found for renally cleared antibiotics (imipenem, piperacillin). Multivariate analysis identified ICG-PDR as the only independent predictor of subtherapeutic concentrations for hepatically or partially eliminated antibiotics (OR = 0.885, 95% CI: 0.807–0.970, *p* = 0.009). In ROC analysis, ICG-PDR demonstrated excellent predictive performance for the entire antibiotic cohort (AUC = 0.853, *p* < 0.001), with a sensitivity of 89.5% and specificity of 72.6% at the optimal cut-off of 20.65%/min. When specifically applied to hepatically cleared antibiotics, it remained a strong predictor (AUC = 0.791, *p* = 0.003), with a cut-off of 21.55%/min yielding a sensitivity of 81.8% and specificity of 71.1%.

**Conclusion:**

AHC is a prevalent phenotype in sepsis and identifies a patient subgroup at high risk for subtherapeutic concentrations of hepatically or partially cleared antibiotics. ICG-derived parameters, particularly ICG-PDR, are robust predictors of inadequate drug exposure, supporting the use of dynamic liver function monitoring to optimize antibiotic dosing in septic patients.

**Supplementary Information:**

The online version contains supplementary material available at 10.1186/s12871-025-03534-9.

## Introduction

 In septic patients, liver function exhibits a broad spectrum, ranging from preserved capacity to limited damage manifesting as mild aminotransferase abnormalities, and even to hepatic failure requiring advanced support such as plasma exchange or liver transplantation [1, 2]. Conventional liver function tests, while useful for detecting impairment, fail to identify patients with augmented hepatic clearance (AHC)—a state of enhanced elimination of drugs primarily cleared by the liver, which is increasingly recognized in specific critically ill populations and can lead to subtherapeutic drug concentrations.

The water-soluble fluorescent dye, indocyanine green (ICG), serves as a dynamic tool for assessing hepatic function. After intravenous administration, ICG binds to plasma proteins and is selectively taken up by hepatocytes. It is then excreted unchanged into the bile, without undergoing metabolism or enterohepatic recirculation. This exclusive hepatic elimination, governed primarily by hepatic blood flow and hepatocyte function, makes ICG clearance a valuable surrogate for liver function [3–5]. ICG clearance can be quantified by two primary parameters: the plasma disappearance rate (ICG-PDR), which reflects the velocity of clearance, and the retention rate at 15 min (ICG-R15), representing percentage of the initially injected ICG dose that remains in the bloodstream 15 min after its intravenous administration. It is important to note that these two parameters reflect opposite aspects of hepatic clearance: a lower ICG-PDR indicates impaired clearance capacity, whereas a lower ICG-R15 suggests enhanced clearance function. A normal ICG-R15 is approximately 6%, with a suggested reference range of 6–12% [6–8]. Consequently, in this study, we define AHC as an ICG-R15 value below 6%, indicating a hepatic clearance rate exceeding the normal upper limit. This definition is supported by prior clinical studies.

The liver’s role in drug clearance involves a complex interplay of blood flow, transporter uptake, and enzymatic metabolism. This dynamic functional capacity is not captured by standard biomarkers like bilirubin or aminotransferases. Enhanced drug clearance has been previously reported in conditions such as cystic fibrosis and pregnancy, often linked to increased hepatic perfusion [9, 10]. For instance, up to 80% of cystic fibrosis patients exhibited subtherapeutic voriconazole concentrations, partly attributable to rapid clearance [11]. Genotypic variations, such as the ultrafast metabolizer phenotype of CYP2C19, further complicate this landscape [12]. Despite its potential impact, the prevalence and significance of AHC in sepsis have received limited attention.

Among the available methods for dynamic liver assessment—including lidocaine metabolism [13], breath tests [14, 15], and scintigraphy—ICG clearance stands out due to its safety, rapidity, and bedside applicability [16, 17]. Both ICG-PDR and ICG-R15 have been used to predict postoperative liver failure and outcomes in critically ill patients [18]. In a previous study, we demonstrated that impaired hepatic clearance, indicated by an ICG-PDR below 17.7%/min, was associated with a high probability of supratherapeutic linezolid exposure [19]. This finding directly prompted the current hypothesis: that AHC may conversely lead to subtherapeutic antimicrobial concentrations, potentially compromising therapeutic efficacy. The present study was therefore designed to investigate this relationship.

The present study was designed with two primary objectives: (1) to evaluate the impact of AHC, defined by ICG clearance, on trough concentrations of commonly used antimicrobials (imipenem/cilastatin, piperacillin/tazobactam, voriconazole, linezolid, vancomycin, cefoperazone/sulbactam, and teicoplanin); and (2) to examine its association with 28-day mortality in septic patients.

## Methods

### Patients and study design

This prospective observational study was conducted in the general intensive care unit (ICU) of Nanjing Drum Tower Hospital. Patients admitted to the ICU and diagnosed with sepsis over a two-month period in 2024 were enrolled. The study was carried out in accordance with the ethical principles outlined in the Declaration of Helsinki (2024 revision). Ethical approval was obtained from the Human Research Ethics Committee of Nanjing Drum Tower Hospital (Approval No: 2024-119-02). All patients or their representatives gave written informed consent for the use of their data.

Exclusion criteria were: (1) patients under 18 years of age; (2) pregnancy or breastfeeding; (3) presence of biliary tract obstruction; (4) presence of chronic liver disease; (5) presence of hyperthyroidism or iodine allergy (ICG contains iodine).

Clinical and biological assessment was performed on ICU admission including gender, age, mean arterial pressure (MAP), lactate (Lac), aspartate aminotransferase (AST), alanine aminotransferase (ALT), total bilirubin (TB), direct bilirubin (DB), γ-glutamyl transferase (γ-GGT), lactate dehydrogenase (LDH), prothrombin time (PT), activate partial thromboplastin time (APTT), international normalized ratio (INR), fibrinogen (Fib), blood urea nitrogen (BUN), creatinine clearance rate (CCr). The sequential organ failure assessment (SOFA) score, acute physiology and chronic health evaluation II (APACHE II) score were assessed within 24 h of ICU admission.

#### ICG clearance test

The ICG clearance test was conducted at the bedside using the LiMON monitoring system (PulsioFlex, Munich, Germany), as previously described [19]. In accordance with our clinical protocol, an initial ICG clearance test was performed for each patient upon hospital admission. Patients were categorized based on their admission ICG-R15 levels, and the impact of these classifications on demographic characteristics and clinical outcomes was analyzed. Whenever a patient required the initiation of a new hepatically cleared antimicrobial agent due to new microbiological evidence or clinical deterioration—a subsequent ICG test was conducted. Subsequent ICG tests were accompanied by simultaneous assessments of illness severity scores (SOFA, APACHE II), vital signs, routine laboratory parameters, and a trough concentration of the newly administered antibiotic measured after it reached steady state. This approach ensured that each ICG assessment was paired with a comprehensive clinical and pharmacokinetic profile. Although individual patients could contribute multiple independent “ICG–drug pairs” to the pharmacokinetic analysis, all cohort-level analyses (such as mortality assessment) were performed on a per-patient basis using data collected at the time of admission.

#### Measurement of CCr

Renal function was assessed by measured 24 h creatinine clearance upon ICU admission. Utilizing the indwelling urinary catheters present in all patients, a complete 24 h urine collection was initiated at a standardized time. The collection began after complete bladder drainage and discarding of the initial urine. The total volume of urine collected over the ensuing 24 h was precisely recorded. Venous blood was drawn during the collection period to determine the serum creatinine concentration. The CCr was then calculated using the standard formula:

$$\begin{aligned} \mathrm{CCr}=&\text{urinary creatinine}\,(\upmu\mathrm{mol}/\mathrm{L})\\&\times\text{total volume of urine in}\,24\,\mathrm{h}\,(\mathrm{L})\\&/\text{serum creatinine concentration}\,(\upmu\mathrm{mol}/\mathrm{L})\\&\times1.44 \end{aligned}$$

#### Antibiotic administration and concentration monitoring

Antibiotic dosing regimens were determined by the attending physician based on standard institutional protocols (detailed in Supplementary Table 1). All beta-lactam antibiotics were administered via intermittent infusion. As part of routine clinical care, therapeutic drug monitoring (TDM) was performed for the studied antimicrobials. Trough concentrations, defined as antibiotic levels measured in the serum after the sixth dose and prior to the next administration, were obtained to guide dosing, and these data were analyzed in the present study.

### Outcomes

The primary endpoint of this study was the incidence of subtherapeutic antibiotic concentration, and the secondary endpoint was 28-day all-cause mortality.

### Definitions of AHC and subtherapeutic drug concentrations

#### AHC

Although AHC lacks a universally standardized definition, it is commonly assessed by ICG-R15, with normal values typically ranging from 6% to 12% [6–8]. In this study, we defined AHC as an admission ICG-R15 value of less than 6%.

#### Subtherapeutic antibiotic concentrations

For β-lactams, subtherapeutic antibiotic concentrations is defined as antibiotic susceptibility breakpoint of enterobacteriaceae bacteria according to the European Committee on Antimicrobial Susceptibility Testing (EUCAST) 2019 guidelines (imipenem/cilastatin<1 mg/L, piperacillin / tazobactam<8 mg/L, cefoperazone/sulbactam<16 mg/L) [20]. As for voriconazole, linezolid, vancomycin and teicoplanin, subtherapeutic concentrations were considered to be below literature target concentrations of voriconazole and linezolid < 2 mg/L [21, 22], vancomycin and teicoplanin < 15 mg/L [23, 24].

### Statistical analysis

All statistical analyses were performed using SPSS Statistics (Version 21.0). Continuous data with non-normal distribution were expressed as median and interquartile range (Q1, Q3), while categorical data were presented as counts and percentages (n, %). The Kruskal-Wallis H test was employed for multiple group comparisons of continuous variables, with post-hoc pairwise comparisons conducted to identify specific inter-group differences. Categorical variables were compared using the Chi-square test. Correlations between ICG clearance parameters (ICG-R15 and ICG-PDR) and antibiotic trough concentrations were assessed using Spearman’s rank correlation coefficient. To identify independent predictors of subtherapeutic concentrations, variables with a significance level of *p* < 0.2 in the univariate analysis were incorporated into a multivariate logistic regression model. The predictive performance of ICG-PDR was further evaluated using receiver operating characteristic (ROC) curve analysis, and the area under the curve (AUC) was calculated. The optimal cutoff value was determined based on the maximum Youden’s index. A two-tailed p-value of less than 0.05 was considered statistically significant.

## Results

### Demographic and baseline clinical Characteristics

A total of 93 patients were included in the final analysis. Based on their initial ICG-R15 values, 46 patients (49.5%) were classified into the AHC group, 16 (17.2%) into the NHC group, and 31 (33.3%) into the IHC group.

The baseline characteristics and outcomes of these groups are summarized in Table 1. The groups were comparable in terms of sex and admission days (all *p* > 0.05). However, significant differences were observed in age and 28-day mortality (both *p* < 0.001).

**Table 1:** Demographic and baseline clinical characteristics

Patients, *n*	AHC ICG-R15 < 6% (*n* = 46)	NHC 6% ≤ ICG-R15 ≤ 12% (*n* = 16)	IHC ICG-R15 > 12% (*n* = 31)	H	*p*
Male sex, n (%)	30 (65.2%)	7 (43.8%)	23 (74.2%)	4.292	0.117
Age, years	65.5 (56.0, 76.3)	80.0 (70.5, 84.0) ^**^	76.0 (69.0, 82.0)^*^	15.245	<0.001
Admission days	21.5 (14.8, 31.5)	21.0 (16.8, 31.3)	19.0 (12.0, 33.0)	0.346	0.841
28 Day mortality, n (%)	5 (10.9%)	2 (12.5%)	15 (48.4%)^*#^	15.766	<0.001

*Abbreviations*: *AHC* augmented hepatic clearance, *NHC* normal hepatic clearance, *IHC* impaired hepatic clearance

*, *p* < 0.05 vs. AHC; **, *p* < 0.01 vs. AHC; #, *p* < 0.05 vs. NHC

Post-hoc pairwise comparisons revealed that patients in both the NHC group and the IHC group were significantly older than those in the AHC group. Regarding the primary outcome of 28-day mortality, the IHC group had a significantly higher mortality rate (48.4%) compared to both the AHC group (10.9%) and the NHC group (12.5%). The difference in mortality between the AHC and NHC groups was not statistically significant.

### Organ function profiles across ICG-R15 strata

A total of 113 ICG measurements were obtained from the 93 enrolled patients. Consequently, all subsequent analyses are based on these 113 independent measurement data points. As shown in Table 1, when stratified by the ICG-R15 value from each measurement, the data points across the AHC, NHC, and IHC groups revealed significant differences in the severity of organ dysfunction.

Compared to the AHC group, data points in the IHC group were consistently associated with a more critical clinical status. These were characterized by significantly higher systemic organ failure scores (SOFA and APACHE II, both *p* < 0.001), a higher incidence of shock (52.6%, *p* < 0.001), and elevated serum lactate levels (*p* < 0.01).

Regarding liver-related metrics, IHC group was also strongly associated with worse hepatic function. This was evidenced by significantly elevated bilirubin levels (TB, DB), markedly impaired coagulation function (PT, APTT, INR), and significantly worsened renal function (BUN, CCr) (all *p* < 0.05). As expected, ICG-PDR demonstrated a significant and progressive decrease from the AHC to the IHC groups (Table 2).

**Table 2:** Organ function characteristics stratified by hepatic clearance groups

Variables	AHC ICG-R15 < 6% (*n* = 56)	NHC 6% ≤ ICG-R15 ≤ 12% (*n* = 19)	IHC ICG-R15 > 12% (*n* = 38)	H	*p*
SOFA, Median (Q1,Q3)	5.0 (3.0, 7.0)	8.0 (4.0, 10.0)	8.0 (6.0, 12.3)^***^	17.477	< 0.001
APACHEⅡ, Median (Q1,Q3)	15 (12.0, 21.0)	17.0 (14.0, 23.0)	24.5 (16.8, 32.0)^***^	20.008	< 0.001
MAP, Median (Q1,Q3)	89.0 (81.0, 96.5)	84.0 (78.0, 96.0)	81.0(73.8, 87.3)^*^	8.628	0.013
Shock, n (%)	8 (14.3%)	6 (31.6%)	20(52.6%) ^**^	14.032	< 0.001
Lac, Median (Q1,Q3)	1.0 (0.8, 1.4)	1.6 (1.2, 3.0)^**^	1.9 (1.3, 3.2)^***^	25.363	< 0.001
AST, Median (Q1,Q3)	24.6 (18.2, 41.9)	36.9 (17.7, 66.3)	34.9 (23.7, 59.9)	4.833	0.089
ALT, Median (Q1,Q3)	22.2 (12.2, 43.5)	34.3 (18.6, 47.5)	22.5 (15.5, 51.8)	1.417	0.492
TB, Median (Q1,Q3)	7.1 (5.5, 12.5)	10.9 (7.9, 23.5)	18.4 (10.7, 91.1)^***^	28.557	< 0.001
DB, Median (Q1,Q3)	2.3 (1.6, 2.9)	4.7(2.3, 11.9)^*^	10.2 (4.7, 64.0)^***^	33.494	< 0.001
γ-GGT, Median (Q1,Q3)	37.2 (19.2, 79.7)	35.1 (22.0, 79.4)	51.0 (29.4, 76.1)	1.348	0.510
LDH, Median (Q1,Q3)	266.0 (202.5, 392.8)	331.0 (290.0, 527.0)	378.5 (239.3, 545.3)^*^	8.040	0.018
PT, Median (Q1,Q3)	12.4 (11.8, 13.1)	12.6 (11.9, 13.9)	13.6 (13.0, 16.1)^***^	24.154	< 0.001
APTT, Median (Q1,Q3)	29.5 (26.0, 33.0)	31.4 (26.2, 33.9)	33.6 (29.0, 43.1)^**^	13.023	0.001
INR, Median (Q1,Q3)	1.1 (1.0, 1.1)	1.1 (1.0, 1.2)	1.2 (1.1, 1.4)^*^	24.154	< 0.001
Fib, Median (Q1,Q3)	4.3 (3.3, 5.5)	4.1 (3.0, 6.4)	3.9 (2.2, 5.3)	1.789	0.409
BUN, Median (Q1,Q3)	9.7 (6.5, 14.1)	13.1 (8.4, 17.9)	15.8 (10.7, 21.5)^**^	13.045	0.001
CCr, Median (Q1,Q3)	77.7 (34.5, 129.3)	43.4 (6.8, 68.2)^*^	27.3 (0.0, 76.5)^**^	15.327	< 0.001
ICG-PDR, Median (Q1,Q3)	23.5 (21.3, 29.5)	17.0 (16.1, 18.0)^***^	10.6 (8.7, 13.0)^***##^	92.839	≤ 0.001

*Abbreviations*: *AHC* augmented hepatic clearance, *NHC* normal hepatic clearance, *IHC* impaired hepatic clearance, *ALT* Alanine Aminotransferase, *APACHE II* Acute Physiology and Chronic Health Evaluation II, *APTT* Activate Partial Thromboplastin Time, *AST* Aspartate Aminotransferase, *BUN* Blood Urea Nitrogen, *CCr* Creatinine Clearance Rate, *DB* Direct Bilirubin, *Fib* Fibrinogen, *ICG-PDR* Indocyanine Green Plasma Disappearance Rate, *ICG-R15* Indocyanine Green Retention Rate at 15 Minutes, *INR* International Normalized Ratio, *Lac* Lactate, *LDH* Lactate Dehydrogenase, *MAP* Mean Arterial Pressure, *PT* Prothrombin Time, *SOFA* Sequential Organ Failure Assessment, *TB* Total Bilirubin, *γ-GGT* γ-Glutamyl Transferase

*, *p* < 0.05 vs AHC; **, *p* < 0.01 vs AHC; ***, *p* < 0.001 vs AHC;##, *p* < 0.01 vs NHC

### ICG clearance and antibiotic exposure

**Fig. 1:**
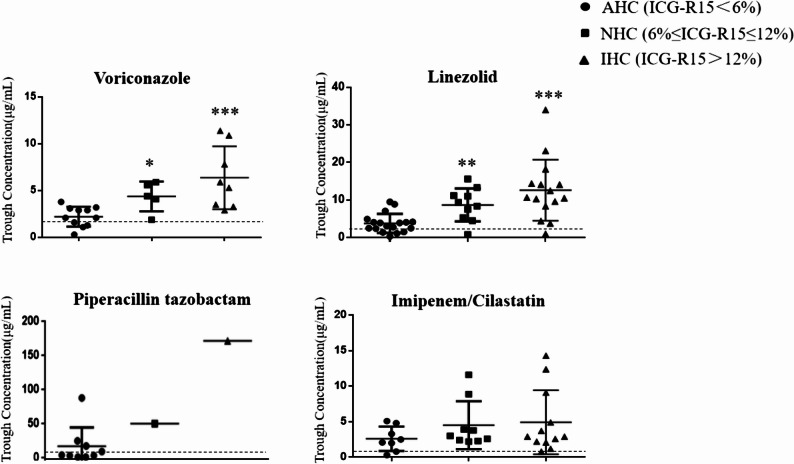

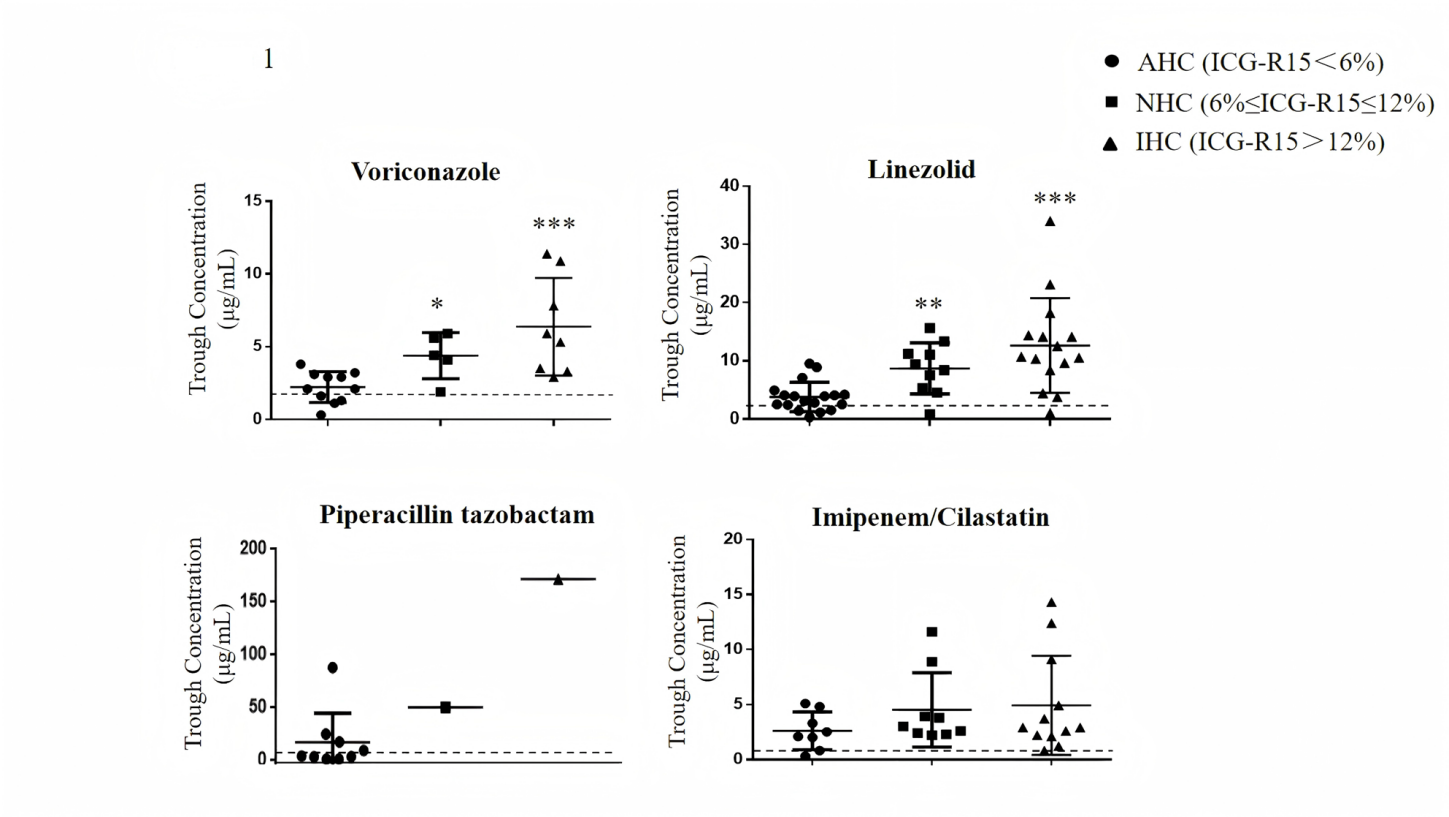
Trough antibiotic concentrations stratified by hepatic clearance group. The dashed horizontal line represents the target concentration. *, *p* < 0.05 vs. AHC; **, *p* < 0.01 vs. AHC; ***, *p* < 0.001 vs. AHC. Abbreviations: AHC, augmented hepatic clearance; NHC, normal hepatic clearance; IHC, impaired hepatic clearance

The relationship between hepatic clearance and antibiotic concentrations was drug-dependent. The incidence of subtherapeutic antibiotic concentrations per drug assay was significantly higher in the AHC group (16/56, 28.5%) compared to the NHC (1/19, 5.2%) and IHC (1/38, 2.6%) groups. This pattern was clearly driven by agents affected by hepatic function: both voriconazole (primarily metabolized by the liver) and linezolid (partially cleared by the liver) exhibited significantly lower trough concentrations in the AHC group, with 20.8% and 14.0% of patients falling below the target, respectively. Their concentrations showed a significant correlation with ICG-PDR and ICG-R15 (Fig. 1). In contrast, the concentrations of the renally cleared antibiotics imipenem and piperacillin demonstrated no correlation with ICG-derived measures of liver function.

**Fig. 2:**
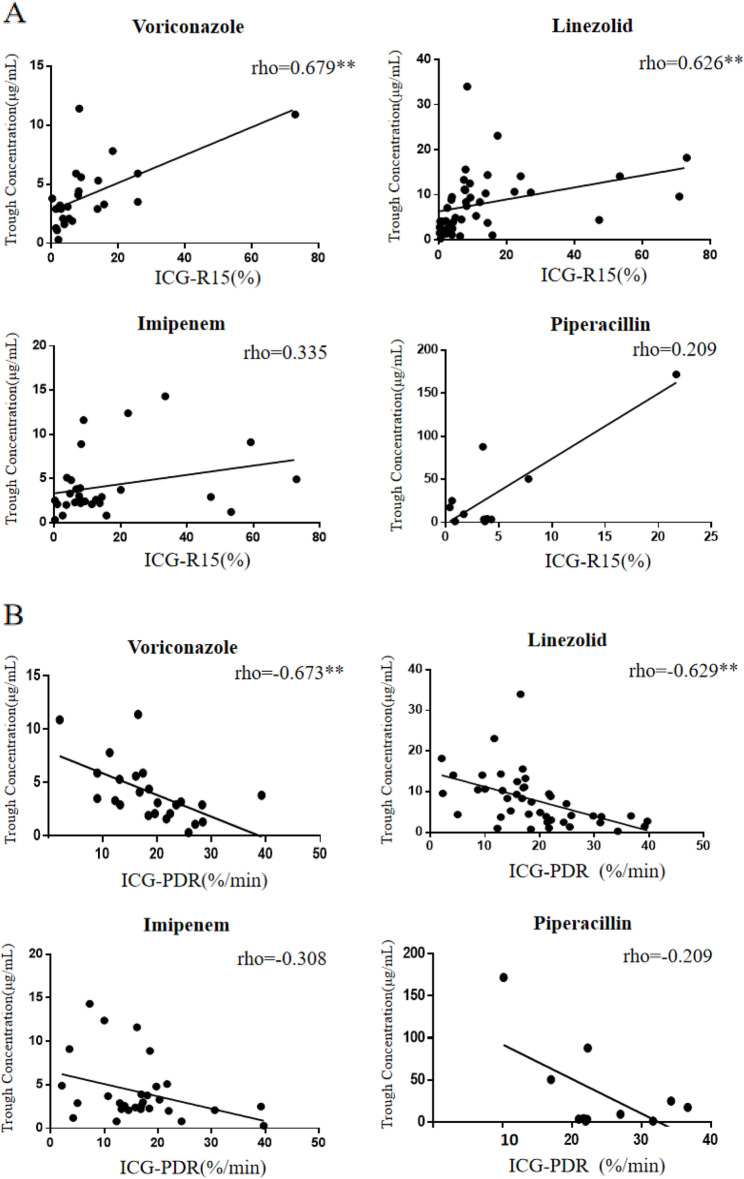

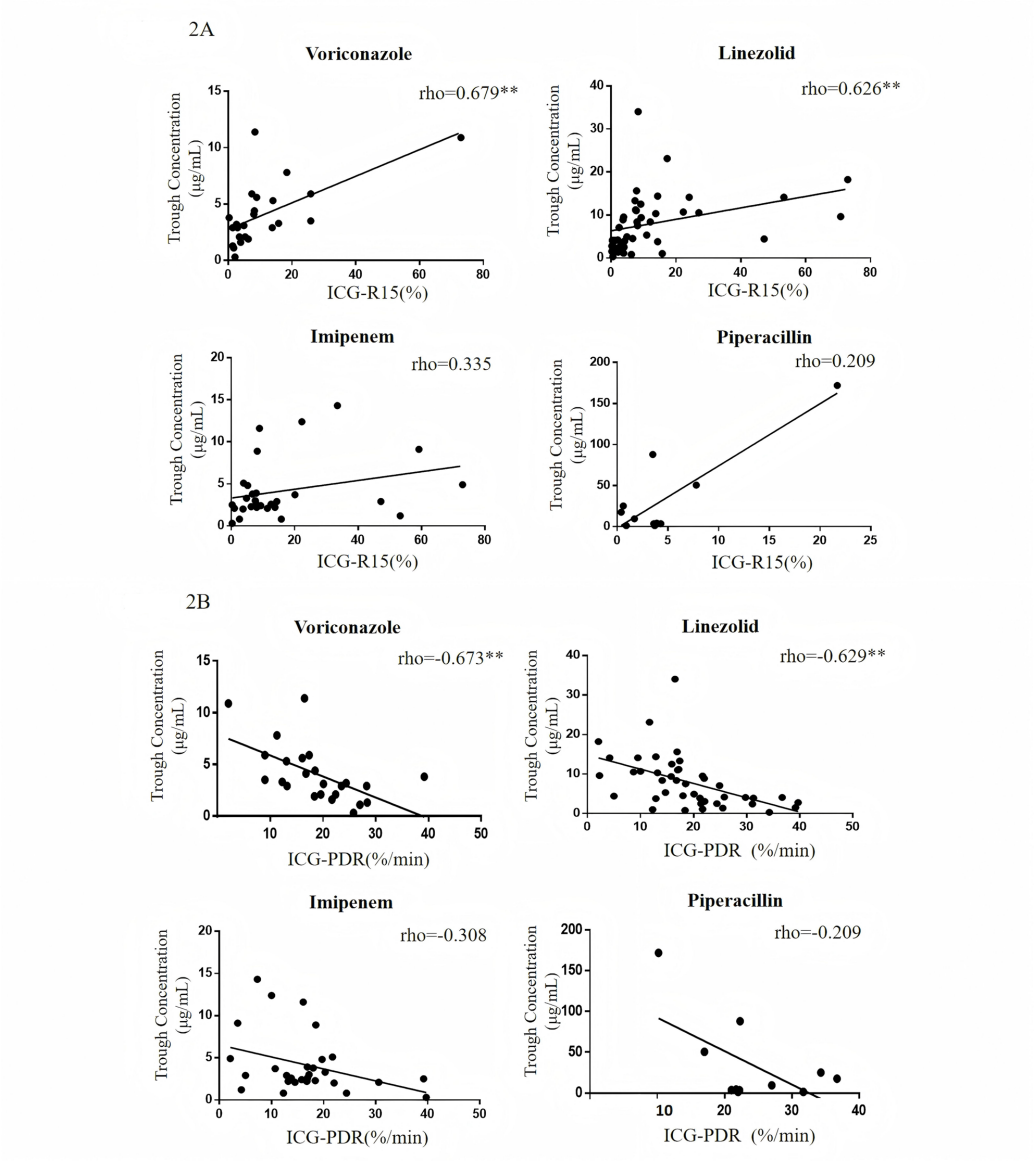
**A** Correlation between trough antibiotic concentration ICG-R15. **, *p* < 0.01. Abbreviations: ICG-R15: indocyanine green retention ratio after 15 min. **B** Correlation between trough antibiotic concentration and ICG-PDR. **, *p* < 0.01. Abbreviations: ICG-PDR: indocyanine green-plasma disappearance rate

Correlations between ICG-R15 and trough antibiotic concentrations were assessed using bivariate analysis and Spearman’s rank test. Voriconazole showed a significant positive correlation (*r* = 0.679), as did linezolid (*r* = 0.626). In contrast, correlations for imipenem (*r* = 0.335) and piperacillin (*r* = 0.209) were not significant (Fig. 2A). Similarly, correlations with ICG-PDR were significantly negative for voriconazole (*r* = -0.673) and linezolid (*r* = -0.629), while no significant correlation was observed for imipenem (*r* = -0.308) or piperacillin (*r* = -0.209) (Fig. 2B).

### Independent predictors of subtherapeutic antibiotic concentration

Univariate analysis was performed to identify factors associated with subtherapeutic concentrations of hepatically eliminated antibiotics. Six variables met the pre-specified threshold of p < 0.2 for inclusion in the multivariate model: SOFA score, lactate, PT, INR, ICG-PDR, and ICG-R15.

In the subsequent multivariate logistic regression analysis, ICG-PDR emerged as the only independent predictor of subtherapeutic concentrations for hepatically eliminated antibiotics (β = -0.122, Wald = 6.844, p = 0.009). The odds ratio for ICG-PDR was 0.885 (95% CI: 0.807–0.970), indicating that each unit increase in ICG-PDR was associated with an 11.5% reduction in the odds of subtherapeutic exposure of these hepatically cleared drugs.

While SOFA score showed significance in univariate analysis for hepatically eliminated antibiotics (OR = 1.316, 95% CI: 1.023–1.694, p = 0.032), it was not retained as an independent predictor in the final multivariate model. Other clinical variables, including APACHE II, shock status, lactate, and conventional liver function tests, did not demonstrate significant associations with subtherapeutic concentrations of hepatically eliminated antibiotics in either univariate or multivariate analyses (Table 3). 

**Table 3:** Univariate and multivariate analysis of predictors for subtherapeutic concentrations of hepatically eliminated antibiotics

	Univariate		Multivariate			
	OR(95%CI)	*p*	β	Wald	OR(95%CI)	*p*
SOFA	1.316(1.023–1.694)	0.032				
APACHEⅡ	1.055(0.963–1.155)	0.252				
Shock	0.366(0.071–1.899)	0.232				
Lac	1.868(0.762–4.581)	0.172				
AST	0.997(0.985–0.997)	0.628				
ALT	0.994(0.979,1.009)	0.441				
TB	1.004(0.990–1.018)	0.579				
DB	1.006(0.985–1.028)	0.562				
Alb	1.004(0.847–1.190)	0.964				
γ-GGT	0.997(0.990–1.003)	0.318				
LDH	1.001(0.997–1.005)	0.594				
PT	1.418(0.862–2.333)	0.169				
APTT	0.988(0.951–1.027)	0.542				
INR	54.457(0.183-16182.327)	0.169				
Fib	1.201(0.799–1.806)	0.377				
BUN	0.968(0.891–1.051)	0.433				
CCr	0.966(0.989–1.003)	0.230				
ICG-PDR	0.885(0.807–0.970)	0.009	-0.122	6.844	0.885(0.807–0.970)	0.009
ICG-R15	1.211(1.001,1.464)	0.049				

*Abbreviations:ALT* Alanine Aminotransferase, *APACHE II* Acute Physiology and Chronic Health Evaluation II, *APTT* Activate Partial Thromboplastin Time, *AST* Aspartate Aminotransferase, *BUN* Blood Urea Nitrogen, *CI* Confidence Interval, *CCr* Creatinine Clearance Rate, *DB* Direct Bilirubin, *Fib* Fibrinogen, *ICG-PDR* Indocyanine Green Plasma Disappearance Rate, *ICG-R15* Indocyanine Green Retention Rate at 15 min, *INR* International Normalized Ratio, *Lac* Lactate, *LDH* Lactate Dehydrogenase, *OR* Odds Ratio, *MAP* Mean Arterial Pressure, *PT* Prothrombin Time, *SOFA* Sequential Organ Failure Assessment, *TB* Total Bilirubin, *γ-GGT* γ-Glutamyl Transferase

In contrast, for renally excreted antibiotics, multivariate analysis identified CCr)as the only independent predictor of subtherapeutic concentrations (OR = 0.983, 95% CI: 0.971–0.997, *p* = 0.013). No other clinical variables showed a significant association in the analysis of this drug class (Supplementary Table 2).

### Predictive performance of ICG-PDR for subtherapeutic antibiotic concentrations

**Fig. 3:**
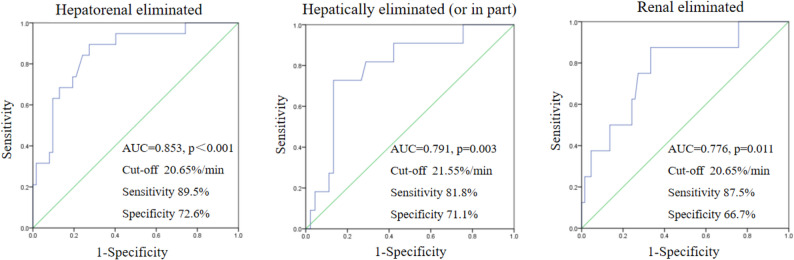

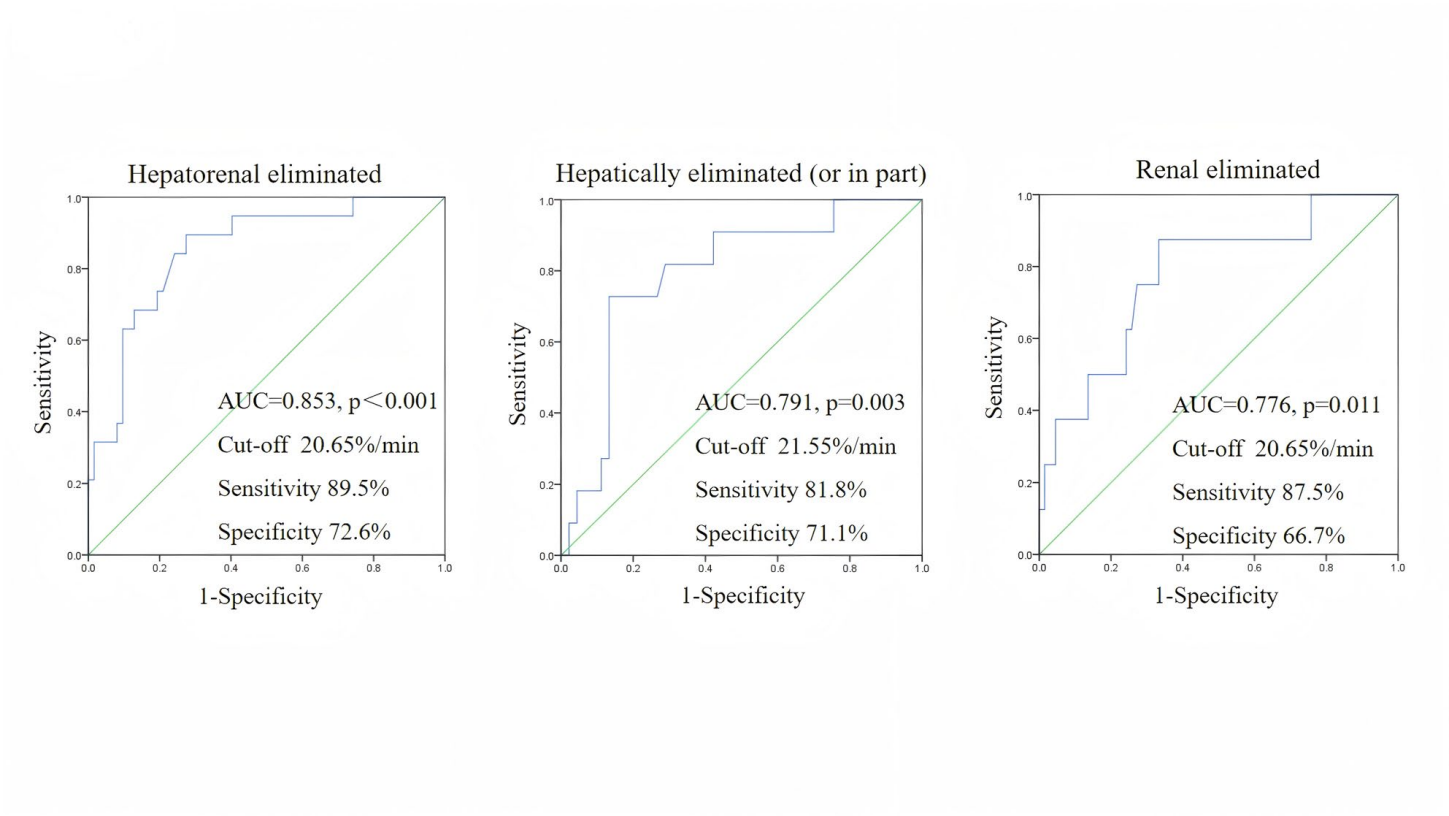
ROC curves of ICG-PDR for predicting subtherapeutic antibiotic concentrations. Abbreviations: AUC, area under the curve; ICG-PDR, indocyanine green plasma disappearance rate; ICG-R15, indocyanine green retention rate at 15 minutes; ROC, receiveroperating characteristic

ROC analysis was performed to evaluate the predictive value of ICG-PDR for subtherapeutic antibiotic concentrations, with analyses stratified by drug elimination route (Fig. 3).

When applied to the entire antibiotic cohort, ICG-PDR demonstrated excellent predictive performance, with an AUC of 0.853 (*p* < 0.001). At the optimal cut-off value of 20.65%/min, it achieved a sensitivity of 89.5% and a specificity of 72.6%.

When the analysis was restricted specifically to antibiotics that are solely or partially cleared by the liver, ICG-PDR remained a strong and significant predictor (AUC = 0.791, *p* = 0.003). The optimal cut-off value for this group was 21.55%/min, yielding a sensitivity of 81.8% and a specificity of 71.1%.

Notably, for antibiotics primarily eliminated by renal excretion, ICG-PDR still showed significant predictive capability, albeit with a comparatively lower performance (AUC = 0.776). The optimal cut-off value derived for this renal clearance subgroup was 20.65%/min, which was identical to that of the overall cohort. At this cut-off, it achieved a sensitivity of 87.5% and a specificity of 66.7%. 

### The correlation between ICG-PDR and hepatic/renal function parameters

**Fig. 4:**
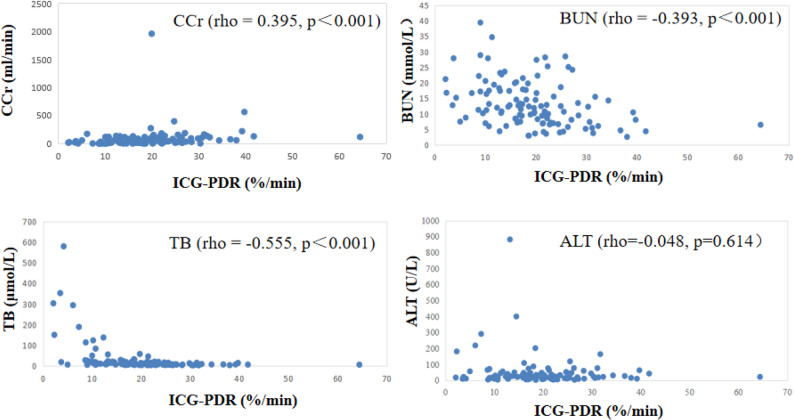

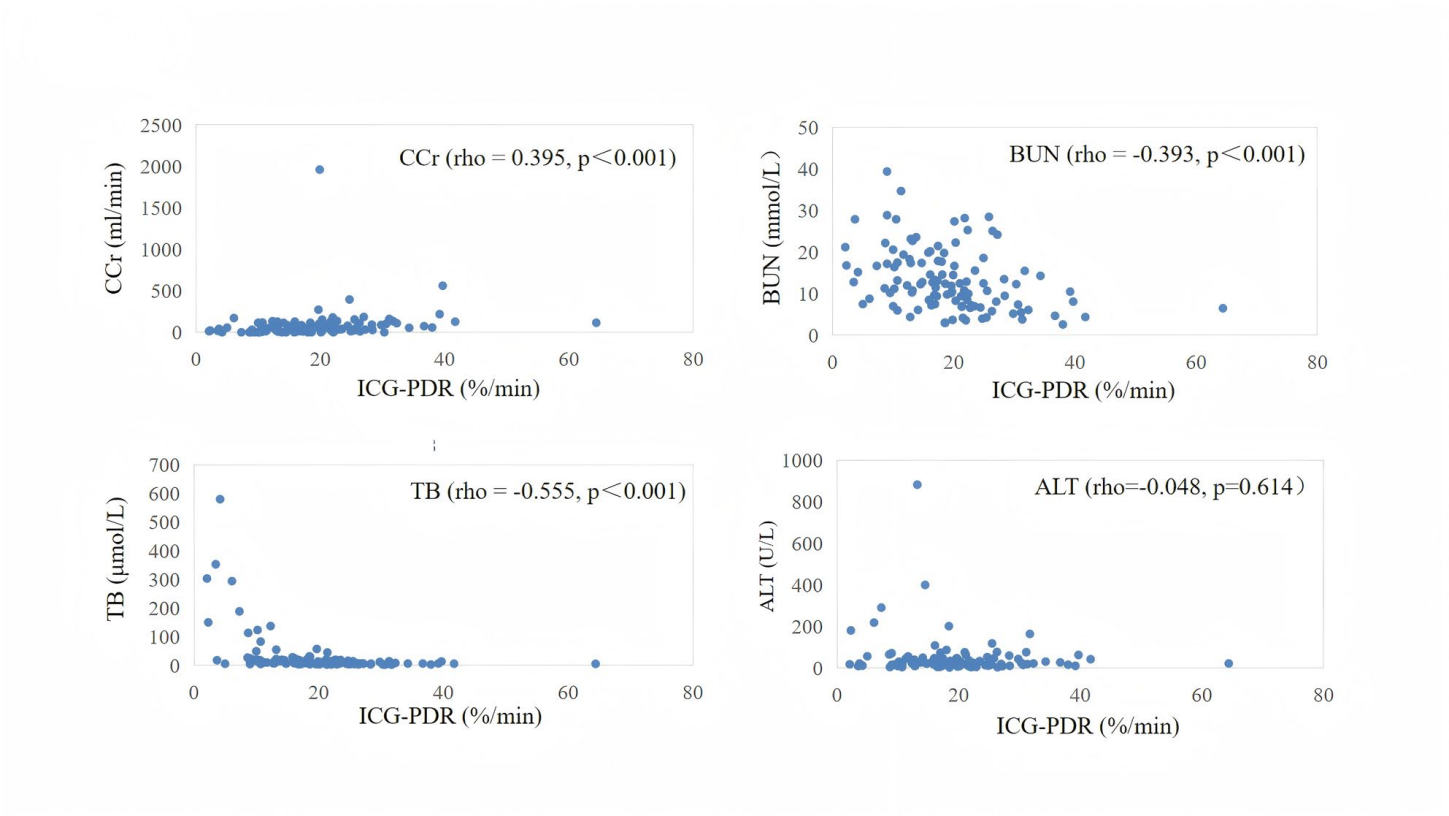
Scatter plots depicting the correlation between ICG-PDR and hepatic/renal function parameters. No regression lines are fitted due to the non-normal distribution of the data, as assessed by non-parametric tests. Abbreviations: ALT, alanine aminotransferase; BUN, blood urea nitrogen; CCr, creatinine clearance rate; ICG-PDR, indocyanine green plasma disappearance rate; TB, total bilirubin

ICG-PDR showed a significant correlation with TB (rho = -0.555, *p*＜0.001), BUN (rho = -0.393, *p*＜0.001), CCr (rho = -0.395, *p*＜0.001). No significant correlations were found between ICG-PDR and ALT (rho = -0.048, *p* = 0.614). As shown in Fig. 4.

## Discussion

This prospective observational study provides novel insights into the prevalence and clinical implications of AHC in critically ill septic patients, as quantitatively assessed by ICG clearance test. Our principal findings are threefold. First, we identified a distinct subgroup of septic patients exhibiting AHC, who demonstrated a significantly higher incidence of subtherapeutic antimicrobial concentrations compared to patients with normal or low hepatic clearance. Second, the correlation between ICG clearance parameters and drug concentrations was elimination-route-specific, being strong and significant for hepatically cleared antibiotics (voriconazole and linezolid) but was not observed for those primarily eliminated renally (imipenem and piperacillin). Third, ICG-PDR was identified as an independent predictor for subtherapeutic exposure after adjusting for key confounders including illness severity scores.

The stratification of our septic cohort by admission ICG-R15 yielded a striking distribution, with nearly half (49.5%) of patients presenting with AHC. This high prevalence establishes AHC not as an uncommon occurrence, but as a predominant physiological phenotype in a significant proportion of septic patients. The high rate of subtherapeutic concentrations (28.5%) in the AHC group underscores a critical and underappreciated facet of sepsis pathophysiology. The hyperdynamic circulation characteristic of sepsis, mediated by systemic vasodilation and increased cardiac output, is a well-established early hemodynamic response. Furthermore, the concomitant systemic inflammatory response, characterized by the release of pro-inflammatory cytokines and mediators, can significantly alter endothelial function and organ perfusion. The hyperdynamic circulation characteristic of sepsis, mediated by systemic vasodilation and increased cardiac output, is a plausible mechanism for AHC, as ICG clearance is highly dependent on hepatic blood flow [25]. Consistent with this mechanism, our findings demonstrate that this state of enhanced clearance has direct and consequential pharmacokinetic effects. The strong negative correlation between ICG-PDR and trough concentrations of voriconazole and linezolid provides compelling evidence that AHC can lead to subtherapeutic exposure, thereby potentially compromising antimicrobial efficacy for specific drug classes. Voriconazole, extensively metabolized by hepatic CYP450 isoenzymes, and linezolid, which undergoes partial hepatic clearance, are particularly vulnerable to this phenomenon [11, 12, 19]. In striking contrast, the lack of correlation for renally excreted drugs like imipenem and piperacillin reinforces the specificity of ICG as a marker of hepatic elimination capacity. This clear dichotomy is crucial for clinicians, as it suggests that AHC primarily poses a risk for drugs with significant hepatic metabolism or clearance—a factor not discernible from conventional static liver function tests.

Interestingly, our analysis reveals that the superior predictive performance of ICG-PDR for subtherapeutic concentrations across all antibiotics (AUC = 0.853), compared to hepatically-cleared drugs alone (AUC = 0.791), aligns with a pattern of coordinated enhancement in both hepatic and renal clearance among patients with AHC. This initially counterintuitive finding can be explained by ICG-PDR serving not merely as a liver-specific metric, but as a sensitive marker of systemic perfusion in sepsis. The early hyperdynamic phase, driven by the inflammatory cascade and resulting in systemic vasodilation and increased cardiac output, enhances blood flow simultaneously to both hepatic and renal systems. Consequently, elevated ICG-PDR identifies patients experiencing globally augmented drug clearance, irrespective of the specific elimination pathway.

The strong correlation between ICG clearance and renal function indicators (CCr, BUN) further supports this concept, suggesting that AHC represents one component of a coordinated systemic hypermetabolic and hyperdynamic response rather than an isolated hepatic phenomenon. This functional coupling between organs, potentially mediated through shared hemodynamic and neuroendocrine pathways, and driven by the underlying inflammatory state, provides the physiological basis for what we term the “integrated clearance phenotype.” This mechanistic insight elucidates why ICG-PDR functions as a universal predictor for antimicrobial underexposure, underscoring its clinical utility in identifying patients at risk across multiple antibiotic classes. These findings collectively substantiate our proposition that ICG-PDR transcends its conventional role as a liver function test. Instead, it provides a unique window into the patient’s overall drug elimination capacity, reflecting a systemic state of augmented clearance that has direct implications for antimicrobial dosing across multiple drug classes.

Our analysis revealed notable differences in both demographic and clinical outcomes across the hepatic clearance spectrum. Patients in the AHC group were significantly younger than those in the NHC and IHC groups, suggesting that preserved physiological reserve in younger individuals may contribute to the augmented clearance phenotype. More importantly, mortality analysis revealed a critical distinction: while the IHC group suffered an exceedingly high mortality rate (48.4%), which aligns with findings from our previous studies establishing this parameter as a marker of organ dysfunction [26], the AHC and NHC groups showed comparable mortality rates (10.9% vs. 12.5%). This pivotal finding demonstrates that although AHC defines a distinct physiological state, it does not independently increase short-term mortality. Instead, the principal hazard associated with AHC is pharmacokinetically mediated, placing this large patient cohort at substantially higher risk of subtherapeutic antimicrobial exposure. This hidden risk of therapeutic failure—undetectable through conventional mortality statistics—represents the central clinical challenge posed by AHC.

Several limitations should be considered when interpreting our findings. First, the single-center observational design may affect the generalizability of our results. Second, although the sample size provided sufficient power for the primary pharmacokinetic analyses, it limited subgroup comparisons and mortality assessments. Methodologically, several factors may have influenced the precision of our pharmacokinetic correlations: ICG measurements can be affected by hematocrit variations in critically ill patients; the timing of ICG tests did not always align with steady-state antibiotic concentrations; and therapeutic drug monitoring was performed using a clinical assay without additional research-level validation. Furthermore, the use of EUCAST breakpoints as therapeutic thresholds, while clinically practical, is less rigorous than the pharmacodynamic target of 100% fT > 4xMIC; this approach was adopted for cohort-wide consistency, as pathogen-specific MICs were unavailable in cases where microbial identification relied on nucleic acid testing. Finally, the lack of serial ICG measurements precluded analysis of hepatic clearance dynamics over time. Future prospective, multi-center studies that incorporate protocolized therapeutic drug monitoring with validated assays, serial ICG assessments, and pathogen-specific MIC data will be essential to validate our proposed ICG cutoffs and to establish the clinical efficacy of ICG-guided dosing.

## Conclusion

In summary, our study shows that liver clearance function identifies two separate risk patterns in sepsis patients. Severely reduced clearance indicates critical illness and is linked to high death rates. On the other hand, patients with augmented clearance face a different kind of risk—not immediate death, but insufficient drug levels due to accelerated elimination. Although patients with augmented clearance had similar survival rates to those with normal function, their drug metabolism patterns were substantially different. This finding suggests we need to change how we evaluate sepsis patients. Regular monitoring of dynamic liver function is essential to detect this hidden risk, allowing for early dosage adjustments in a significant number of sepsis patients who may not respond to standard antibiotic regimens.

## Supplementary Information

Supplementary Material 1: Supplementary Table 1. Summary of Antibiotic Dosing Regimens Based on Renal Function. Supplementary Table 2. Univariate and Multivariate Analysis of Factors Associated with Subtherapeutic Concentrations of Renally Cleared Antibiotics.

## Data Availability

The data are available from the corresponding author upon reasonable request.

## Abbreviations

ALT: Alanine Aminotransferase
APACHE II: Acute Physiology and Chronic Health Evaluation II
APTT: Activate Partial Thromboplastin Time
AST: Aspartate Aminotransferase
AUC: Area under the curve
AHC: Augmented hepatic clearance
BUN: Blood Urea Nitrogen
CCr: Creatinine Clearance Rate
DB: Direct Bilirubin
EUCAST: European Committee on Antimicrobial Susceptibility Testing
Fib: Fibrinogen
γ-GGT: γ-glutamyl transferase
ICG: Indocyanine green
ICG-PDR: Indocyanine Green Plasma Disappearance Rate
ICG-R15: Indocyanine Green Retention Rate at 15 min
ICU: Intensive care unit
INR: International Normalized Ratio
Lac: Lactate
LDH: Lactate Dehydrogenase
MAP: Mean Arterial Pressure
PT: Prothrombin Time
ROC: Receiver operating characteristic
SOFA: Sequential Organ Failure Assessment
TB: Total Bilirubin
