# Supplementary material for: ICG clearance as an indicator of augmented hepatic clearance and subtherapeutic drug concentrations in septic patients
Source: BMC Anesthesiol. 2025 Dec 10;26:36. doi: 10.1186/s12871-025-03534-9 (PMC12801564; doi:10.1186/s12871-025-03534-9)
Supplement: Supplementary file 1 — Supplementary Material 1: Supplementary Table 1. Summary of Antibiotic Dosing Regimens Based on Renal Function. Supplementary Table 2. Univariate and Multivariate Analysis of Factors Associated with Subtherapeutic Concentrations of Renally Cleared Antibiotics. [file 12871_2025_3534_MOESM1_ESM.doc]

**Supplementary Table 1. Summary of Antibiotic Dosing Regimens Based on Renal Function**

| **Antibiotic** | Standard / Loading Dose | Maintenance Dosing (Adjusted by CCr) |
| --- | --- | --- |
| **Imipenem/Cilastatin** |  | **CCr ≥90 mL/min:** 1000 mg every 8h **60≤ CCr <90 mL/min:** 500 mg every 6h **30≤ CCr <60 mL/min:** 500 mg every 8h **15≤ CCr <30 mL/min:** 500 mg every 12h |
| **Linezolid** |  | **600 mg IV every 12h (No renal adjustment required)** |
| **Voriconazole** |  | **4 mg/kg every 12h (No standard renal adjustment; monitor for toxicity)** |
| **Piperacillin/Tazobactam** |  | **CCr ≥20 mL/min:** 4.5 g every 8h **CCr <20 mL/min:** 4.5 g every 12h |
| **Vancomycin** | **6 mg/kg every 12h (for first 24h)** | **CCr ≥130 mL/min:** 1000 mg every 8h **70≤ CCr <130 mL/min:** 1000 mg every 12h **50≤ CCr <70 mL/min:** 500 mg every 8h **30≤ CCr <50 mL/min:** 500 mg every 12h **CCr ≤30 mL/min:** 500 mg daily |
| **Cefoperazone/Sulbactam** |  | **3 g every 8h** In severe biliary obstruction, liver disease, or renal dysfunction: Adjust dose based on TDM |
| **Teicoplanin** | **6 mg/kg administered every 12 hours (a total of 3 doses)** | **CCr ≥80 mL/min:** 6 mg/kg every 24h **30≤ CCr <80 mL/min:** 6 mg/kg every 48h **CCr <30 mL/min:** 6 mg/kg every 72h |

**Abbreviations: CCr, Creatinine Clearance Rate; IV, Intravenous; TDM, Therapeutic Drug Monitoring**

**Supplementary Table 2. Univariate and Multivariate Analysis of Factors Associated with Subtherapeutic Concentrations of Renally Cleared Antibiotics.**

|  | Univariate | | Multivariate | | | |
| --- | --- | --- | --- | --- | --- | --- |
|  | OR（95%CI） | p | β | Wald | OR（95%CI） | p |
| SOFA | 1.436 (1.057-1.952) | **0.021** |  |  |  |  |
| APACHEⅡ | 1.067 (0.960-1.185) | 0.229 |  |  |  |  |
| Shock | 0.220 (0.026-1.892) | **0.168** |  |  |  |  |
| Lac | 2.380 (0.753-7.521) | **0.140** |  |  |  |  |
| AST | 1.000 (0.994-1.007) | 0.954 |  |  |  |  |
| ALT | 0.997 (0.988-1.007) | 0.606 |  |  |  |  |
| TB | 1.002 (0.990-1.015) | 0.696 |  |  |  |  |
| DB | 1.005 (0.984-1.027) | 0.639 |  |  |  |  |
| Alb | 0.892 (0.743-1.071) | 0.221 |  |  |  |  |
| γ-GGT | 0.998 (0.990-1.006) | 0.593 |  |  |  |  |
| LDH | 1.001 (0.997-1.006) | 0.556 |  |  |  |  |
| PT | 1.354 (0.779-2.353) | 0.283 |  |  |  |  |
| APTT | 0.980 (0.942-1.020) | 0.329 |  |  |  |  |
| INR | 31.685 (0.058-17235.001) | 0.282 |  |  |  |  |
| Fib | 1.170 (0.775-1.764) | 0.455 |  |  |  |  |
| BUN | 1.048 (0.929-1.182) | 0.444 |  |  |  |  |
| CCr | 0.983 (0.971-0.997) | **0.013** | -0.017 | 6.154 | 0.983 (0.971-0.997） | 0.013 |
| ICG-PDR | 0.901 (0.830-0.977） | **0.012** |  |  |  |  |
| ICG-R15 | 1.188 (0.974-1.449) | **0.089** |  |  |  |  |

Abbreviations: ALT, Alanine Aminotransferase; APACHE II, Acute Physiology and Chronic Health Evaluation II; APTT, Activate Partial Thromboplastin Time; AST, Aspartate Aminotransferase; BUN, Blood Urea Nitrogen; CI, Confidence Interval; CCr, Creatinine Clearance Rate; DB, Direct Bilirubin; Fib, Fibrinogen; ICG-PDR, Indocyanine Green Plasma Disappearance Rate; ICG-R15, Indocyanine Green Retention Rate at 15 Minutes; INR, International Normalized Ratio; Lac, Lactate; LDH, Lactate Dehydrogenase; OR, Odds Ratio; MAP, Mean Arterial Pressure; PT, Prothrombin Time; SOFA, Sequential Organ Failure Assessment; TB, Total Bilirubin; γ-GGT, γ-Glutamyl Transferase
